# Orai storeoperated Ca^2+^ entry channels modulate urethral smooth muscle contractility

**DOI:** 10.1080/19336950.2025.2583809

**Published:** 2025-11-05

**Authors:** Bernard T. Drumm, Neha Gupta

**Affiliations:** Smooth Muscle Research Centre, Department of Life & Health Science, Dundalk Institute of Technology, Dundalk, Ireland

**Keywords:** Orai, STIM, SOCE, urethra, smooth muscle, Ca^2+^ channel, store operated, sarcoplasmic reticulum, incontinence

## Abstract

In the European Union, urinary incontinence (UI) affects 45% of adults during their lifetime, representing a major clinical and socio-economic burden. Failure of urethral smooth muscle (USM) to contract normally (hypo or hypercontractility) contributes to UI symptoms such as urine leakage during bladder filling or inability to urinate due to obstruction. Adequate UI treatments are lacking, partially due to a lack in understanding of cellular mechanisms underlying USM contraction. USM contractions rely on Ca^2+^ signaling in urethral smooth muscle cells (USMC), resulting from Ca^2+^ release from internal stores and Ca^2+^ influx from extracellular sources, such as voltage-gated L-type Ca^2+^ channels or store-operated Ca^2+^ entry (SOCE) channels. L-type Ca^2+^ channel inhibitors have inconsistent effects on urethral contractions across species, including humans, and thus solely targeting this pathway may be insufficient to modulate USM contractility. Recent animal experiments suggest SOCE mediated by Orai-STIM proteins is a critical determinant of Ca^2+^ signaling in USMC, maintaining regenerative Ca^2+^ release from internal stores, and thus may be a targetable pathway for influencing USM contractility. In this review, we highlight evidence suggesting SOCE as critical for Ca^2+^ signaling in USMC from multiple species and propose possible mechanisms for how this occurs at the cellular level.

## Introduction

In the European Union (EU), urinary incontinence (UI) can affect ~45% of adults during their lifetime [1], causing involuntary leakage, urgency, incomplete bladder emptying, and overactive bladder (OAB) symptoms [2,3]. Beyond the physical burden, UI contributes to recurrent urinary tract infections, poor sleep, anxiety, and depression, with an estimated annual economic cost to the EU of €69.1 billion in 2023, projected to exceed €100 billion by 2030 [1]. UI arises from disrupted coordination between both bladder and urethral smooth muscle cells (SMC). During filling, bladder SMC relax to accommodate urine while urethral SMC contract to close the urethral outlet, thus maintaining continence; during voiding, this pattern reverses [4]. Failure of this reciprocal system underlies UI, yet the molecular mechanisms that regulate this are still not fully understood.

**Figure 1.**
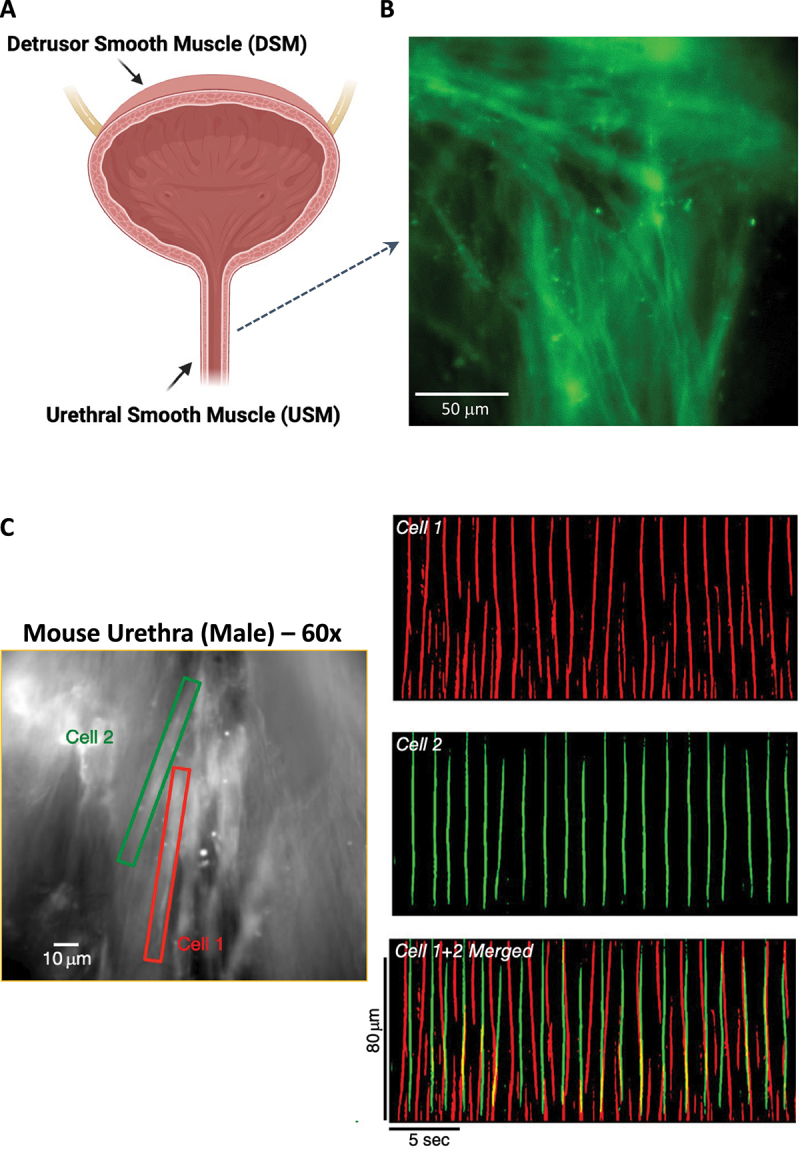
Asynchronous activity of urethral smooth muscle bundles. A. Lower urinary tract comprising detrusor smooth muscle (DSM) of bladder and urethral smooth muscle (USM) of the internal urethral sphincter. B. USM layers of urethral smooth muscle cells (USMC) arranged in an interlaced basket weave with roughly circular and longitudinal orientation (adapted with permission from [6]). C. Still image from male urethral tissue from a mouse expressing a smooth muscle specific Ca^2+^ indicator (GCaMP3). Two cells are highlighted in red (Cell 1) and green (Cell 2) and spontaneous Ca^2+^ activity displayed as spatiotemporal maps (STMs) on the right. Merging the STMs shows that USMC activity is asynchronous, even when cells are adjacent to each other (adapted with permission from [7]).

The walls of the internal urethral sphincter are composed of layers of urethral smooth muscle cells (USMC) arranged in circular and longitudinally orientated bundles [5] (Figure 1(A,B)). During bladder filling, USMC contract asynchronously across multiple bundles (Figure 1(C)), summating to generate myogenic tone to occlude the urethral orifice [8,9], preventing bladder leakage and maintaining urinary continence [10]. At the onset of micturition, urethral smooth muscle (USM) relaxes (in response to parasympathetic release of nitric oxide [11]) to allow passage of urine to the exterior. Currently, there is a lack of noninvasive interventions to treat urethral malfunctions [2,12], and USM is relatively understudied compared to the skeletal muscle of the external urethral sphincter (rhabdosphincter). However, there is increasing awareness that contractions of USM and not the rhabdosphincter provide the greatest contribution to urethral closure pressure to maintain continence during bladder filling [13–16]. Thus, the relevance of USM in urinary tract disorders such as stress incontinence is increasingly clinically recognized [13], and means of targeting molecular mechanisms controlling USMC contraction and relaxation are of increasing interest for urinary tract physiologists.

Like all smooth muscle, contractions and relaxations of USMC are controlled by intracellular Ca^2+^ ([Ca^2+^]_i_) levels [17,18]. Increased [Ca^2+^]_i_ activates the myosin-light chain kinase pathway, leading to phosphorylation of myosin filaments allowing interaction with actin proteins that initiates contraction of smooth muscle [19]. Conversely, the relaxation of USMC is due to an appropriate decrease in [Ca^2+^]_i_. In USMC, [Ca^2+^]_i_ is tightly regulated by Ca^2+^ release and sequestration via internal stores in the sarcoplasmic reticulum (SR) [7,20], lysosomes [21], and Ca^2+^ influx from the extracellular space [7,20]. USMC Ca^2+^ transients arise spontaneously from multiple firing sites in individual USMC, and are the result of SR Ca^2+^ release via inositol-tri-phosphate receptors (IP_3_Rs) and ryanodine receptors (RyRs), but predominately IP_3_R1 [7]. Ca^2+^ release in USMC occurs spontaneously, but can be enhanced when intracellular IP_3_ levels are increased by binding of excitatory agonists and neurotransmitters (noradrenaline, norepinephrine, phenylephrine) to adrenergic alpha1 receptors [22]. Activation of Gq coupled alpha1 receptors leads to synthesis of phospholipase C (PLC) which cleaves plasma membrane located phosphatidylinositol 4,5-bisphosphate (PIP_2_) to IP_3_ and diacylglycerol (DAG). IP_3_ causes further SR Ca^2+^ release by activating IP_3_Rs. The SR Ca^2+^ store is maintained by Ca^2+^ refilling from the cytoplasm via SR Ca^2+^-ATPase (SERCA) proteins [7]. SR Ca^2+^ release in USMC can be modified from lysosomal Ca^2+^ release via transient potential melastatin 1 (TRMPM1) which induce SR Ca^2+^ release in USMC via Ca^2+^ induced SR Ca^2+^ release (CICR) on RyRs [21].

Historically, Ca^2+^ influx through voltage-dependent Ca^2+^ channels (specifically L-type Ca^2+^ channels) was thought to be the primary extracellular Ca^2+^ source for USMC [5,9,23,24]. However, pharmacological inhibitors of L-type Ca^2+^ channels (nifedipine, nicardipine, verapamil) have disparate effects on USMC activity and contractions [7,23,25]. L-type Ca^2+^ channel inhibitors have inconsistent effects on urethral contractions evoked by noradrenaline in sheep [26], rabbit [27] and cats [28]. Importantly, nifedipine fails to significantly affect urethral tone measured in humans [29], this combined with its known hypotensive effects has limited its widespread clinical use for relaxing USM (such as in benign prostatic hyperplasia) [30]. Thus, solely targeting L-type Ca^2+^ channels to modulate USMC activity may not be ubiquitously beneficial for treating incontinence disorders stemming from urethral malfunction. It therefore behooves urinary tract physiologists to investigate other pathways that affect USMC contractility. Previous extensive reviews are available chronicling the ionic apparatus impacting excitability of USMC through activation of Ca^2+^ and voltage dependent K^+^ channels [31], Ca^2+^-activated-Cl^−^ channels [20,32,33] and voltage-dependent Ca^2+^ channels [8,9,20]. In this review, we will summarize more recent work that highlights store-operated-Ca^2+^-entry (SOCE) mediated through Orai channels as an emerging essential pathway for normal USMC function in multiple species.

## Store-operated Ca^2+^ entry (SOCE) and urethral smooth muscle spontaneous activity

**Figure 2.**
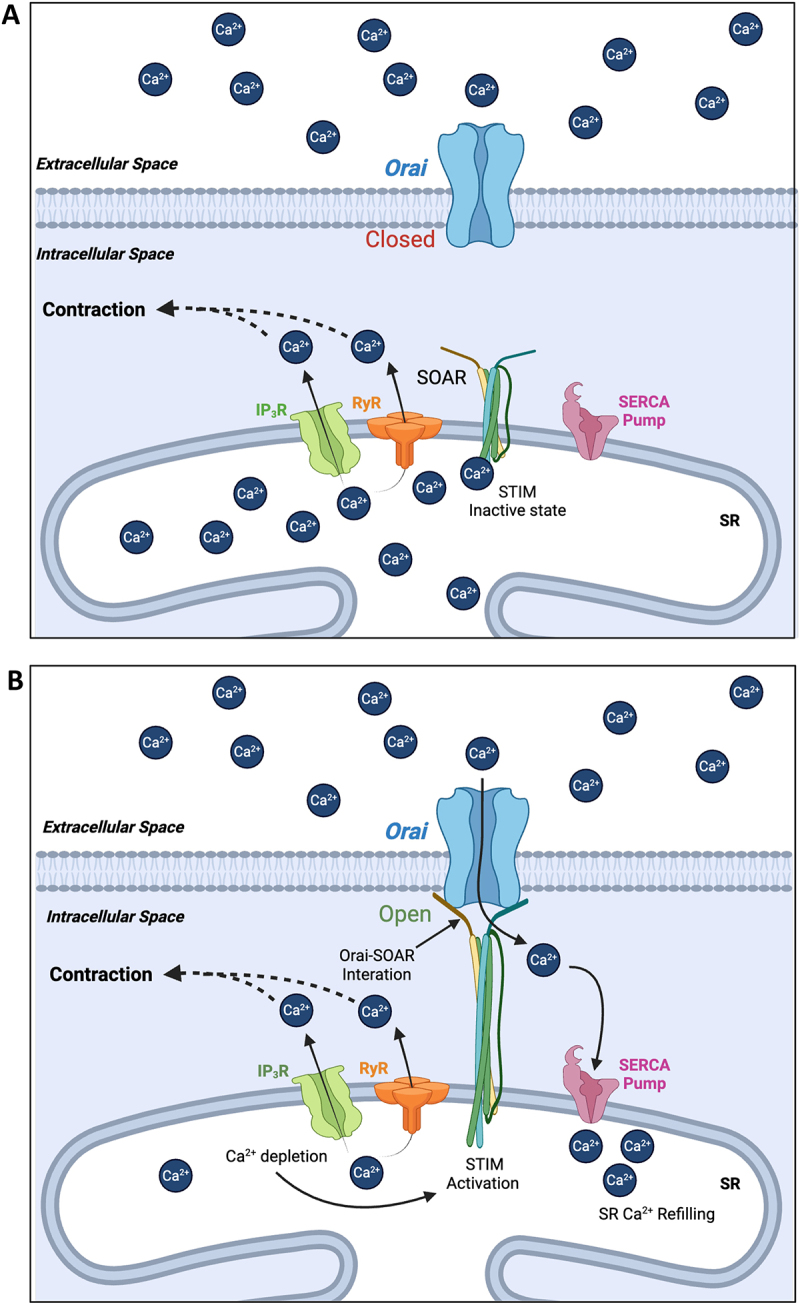
Mechanism of STIM–Orai interactions during store-operated Ca^2+^ entry in smooth muscle. A. When SR Ca^2+^ stores are sufficiently full, Ca^2+^ binds to the luminal binding site of SR membrane bound STIM proteins, preventing interaction between STIM–Orai–activating regions, (SOAR) and plasma membrane bound Orai channels which keeps Orai closed. B. Upon a fall in SR Ca^2+^ due to Ca^2+^ release (via IP_3_Rs and RyRs), STIM oligomerizes and translocates to the plasma membrane allowing SOAR binding to Orai and opens the channel. Opening of Orai channels allows Ca^2+^ influx into the cytoplasm, which can refill the SR store through SERCA pump pathways. In the case of USMC, Orai mediated Ca^2+^ influx contributes to contraction through possible mechanisms outlined in Figure 5.

As outlined in Figure 2, SOCE is mediated by molecular interactions between stromal interaction molecule (STIM) (of which there are two variants (STIM1 and STIM2)) expressed on the membrane of the internal Ca^2+^ store of the SR [34–38], and a family of Ca^2+^ channels in the plasma membrane, Orai 1–3 [39–42]. Binding of SR Ca^2+^ to luminal interaction sites of STIM (Figure 2(A)) maintains the molecule in a monomeric conformation [43,44]. A fall in [Ca^2+^]_SR_ results in unbinding of Ca^2+^ from luminal STIM sites [45], inducing a structural rearrangement, oligomerization, and translocation to the plasma membrane (Figure 2(B)), such that the C-terminal binding hands of STIM (STIM-Orai-activating-region, SOAR [46,47]), bind to Orai channels in the plasma membrane [48–54]. Consequential interaction of STIM and Orai opens the Orai channel, facilitating Ca^2+^ influx into the cytoplasm, increasing [Ca^2+^]_i_, which refills the SR Ca^2+^ load by Ca^2+^ sequestration from the cytoplasm via SERCA pumps [36,37,39–41,55,56].

Contractions of USM bundles are generated by firing of intracellular Ca^2+^ transients in USMC. These Ca^2+^ transients are spatially and temporally diverse, ranging from discrete and brief transients lasting ms to propagating intracellular waves that last many seconds [7,8]. USMC Ca^2+^ transient firing occurs asynchronously across multiple cells within a bundle (Figure 1(C)), and correlate with asynchronous contractions of USMC bundles, which when summated across the tissue manifest as uniform myogenic tone [57]. USMC Ca^2+^ transients occur spontaneously and frequently, with some cells firing hundreds of Ca^2+^ transients every minute across up to 5–8 sites per cell. Such an incredible and relatively constant loss of Ca^2+^ from the SR would necessitate an efficient mechanism for SR Ca^2+^ store refilling (and/or to maintain sufficient cytosolic Ca^2+^ levels to maintain an excitable medium for Ca^2+^ transient initiation or propagation [58]) to maintain regenerative Ca^2+^ release over extended periods. In other smooth muscle tissues, SOCE through Orai is important to maintain SR Ca^2+^ content to sustain regenerative spontaneous activity and responses to agonists [59–67], and the following sections will outline evidence for such a mechanism in USM from multiple species.

## Effects of SOCE inhibitors on spontaneous USMC activity

Early evidence for SOCE modulating urethral activity came from electrophysiological recordings of sheep USMC. 10% of isolated sheep USMC exhibit spontaneous transient inward currents (STICs) in voltage clamp (−60 mV), mediated by Ca^2+^-activated-Cl^−^ conductances (presumably activated by intracellular Ca^2+^ transients similar to those observed in rabbit [68] and mice [7]) that were reduced in amplitude and frequency by SKF-96365 and La^3+^, both nonselective SOCE inhibitors [69]. In experiments where USMC Ca^2+^ transients were imaged from male mice expressing a smooth muscle-specific Ca^2+^ reporter (GCaMP3 driven by smooth muscle myosin heavy chain), nifedipine (L-type Ca^2+^ channel inhibitor) failed to affect these spontaneous Ca^2+^ transients [7]. In contrast, the nonselective SOCE inhibitor SKF-96365 and selective Orai channel inhibitor GSK-7975A reduced USMC Ca^2+^ transient firing frequency by more than 50%, as well as reducing their amplitude, duration, and spatial spread [7]. The residual Ca^2+^ events that persisted when SOCE was blocked likely originate solely from Ca^2+^ release via the remaining SR Ca^2+^ load, as all Ca^2+^ events were abolished when the SR was fully depleted with SERCA pump inhibitors (cyclopiazonic acid or thapsigargin) [7].

qPCR analysis of enriched populations of murine USMC revealed expression of all three Orai gene variants, with *Orai1* and *Orai3* expressed at similar levels, and *Orai2* expressed 60% less than *Orai1* and *Orai3* [7]. In HEK293 cells, all three Orai variants are expressed as heteromultimer complexes to coordinate complex intracellular Ca^2+^ signals in the form of repetitive IP_3_R mediated Ca^2+^ oscillations and Ca^2+^ transients [70,71]. Expression of Orai2 and Orai3 within the complex negatively regulates Orai1 opening by enhancing Ca^2+^ dependent inactivation (CDI) of Orai1 [71]. As Orai1 activation mediates the largest Ca^2+^ current of the three variants, enhancement of Orai1 CDI prevents cellular Ca^2+^ overload [71]. As the Ca^2+^ signaling patterns of USMC are varied, composing localized Ca^2+^ release events similar to Ca^2+^ puffs [72,73] or Ca^2+^ sparks [74] as well as large amplitude Ca^2+^ oscillations and waves [7], the expression of Orai2 and Orai3 in these cells could indicate a requirement of finely tuned CDI of Orai1 to prevent USMC from experiencing global and uniform increases in [Ca^2+^]_i._ This would result in over contractile urethral bundles and possible disruption of the urethral sphincter to coordinate relaxations during micturition.

The relative expression of STIM variants in USMC is currently unknown and warrants investigation. While both STIM1 and STIM2 are required for complex Ca^2+^ transients as displayed by USMC [70], and thus both STIM paralogs are likely expressed in these cells, they are regulated differently by [Ca^2+^]_SR_ levels [34], and differentially interact with Orai channels (STIM2 acts as only a partial agonist of Orai compared to STIM1 [53]). Therefore, information on the relative expression of STIM1 and STIM2 may provide insight into how SOCE in USMC coordinates the orchestration of the continuum of complex Ca^2+^ signals in the urethra.

## SOCE via Orai channels contribute to USM contractions

Functional SOCE that impacts USM contractility has been demonstrated in mice and pig. SOCE can be evoked in these tissues by firstly depleting SR Ca^2+^ stores and preventing SR refilling in organ baths containing strips or rings of dissected USM tissues [22]. This is accomplished by incubating tissues in a Ca^2+^ free solution in the presence of a SERCA pump inhibitor (thapsigargin or CPA). This depletes SR Ca^2+^ stores, resulting in maximum interaction between STIM and Orai. In the continued presence of the SERCA pump inhibitor, normal levels of Ca^2+^ can be reintroduced to the organ bath, allowing Ca^2+^ to flow into USMC through now opened Orai channels. Due to the continued blockade of SERCA, the influx of Ca^2+^ cannot be pumped into the SR and thus cytoplasmic Ca^2+^ remains highly elevated, resulting in an exaggerated “overshoot” of contraction relative to baseline, indicative of SOCE [75].

**Figure 3.**
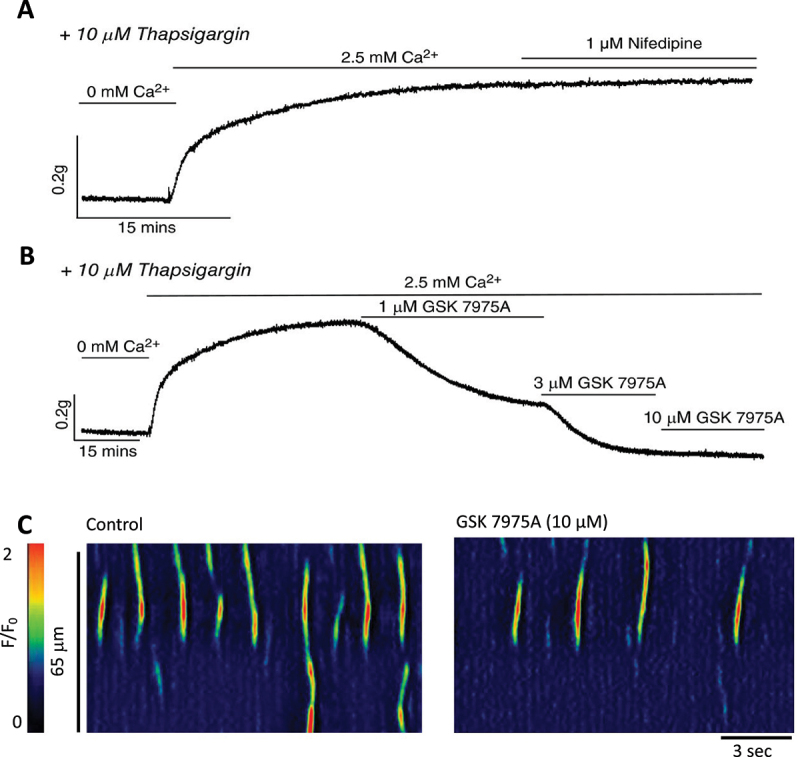
SOCE sustains Ca^2+^ signaling in mouse USM A. SOCE evoked in male mouse USM tissues. Urethral rings were placed in an organ bath to measure isometric tension. Upon SR depletion with 0 mM Ca^2+^ solution and the SERCA pump inhibitor thapsigargin, reintroduction of normal extracellular Ca^2+^ (2.5 mM) in the continued condition of SERCA inhibition, a massive “overshoot” in contraction was observed and this was resistant to inhibition of L-type Ca^2+^ channels by nifedipine. B. SOCE contraction overshoot in male mouse urethra dose-dependently reduced by the Orai inhibitor GSK-7975A. C) GSK-7975A reduces spontaneous Ca^2+^ events in USMC (measured from a cell within an intact mouse USM sheet) that underlie asynchronous activity that summate to form myogenic tone (adapted with permission from [7]).

When this SOCE evoking protocol was applied to mouse and pig USM tissues, a huge SOCE “overshoot” contraction was evident, with the overshoot almost 3 times larger than basal tone in mice [7], and doubled normal tone in pig USM [25]. Importantly, while inhibiting L-type Ca^2+^ channels with nifedipine had no effect on the SOCE overshoot in mice, the Orai inhibitor GSK-7975A dose-dependently reduced the overshoot [7] (Figure 3(A,B)). In addition, GSK-7975A reduced Ca^2+^ transients in mouse USMC by 50% (Figure 3(C)), reduced contractions evoked by the adrenergic agonist phenylephrine [7] and post-junctional neurally evoked contractions elicited by electrical field stimulation by >50% [76].

**Figure 4.**
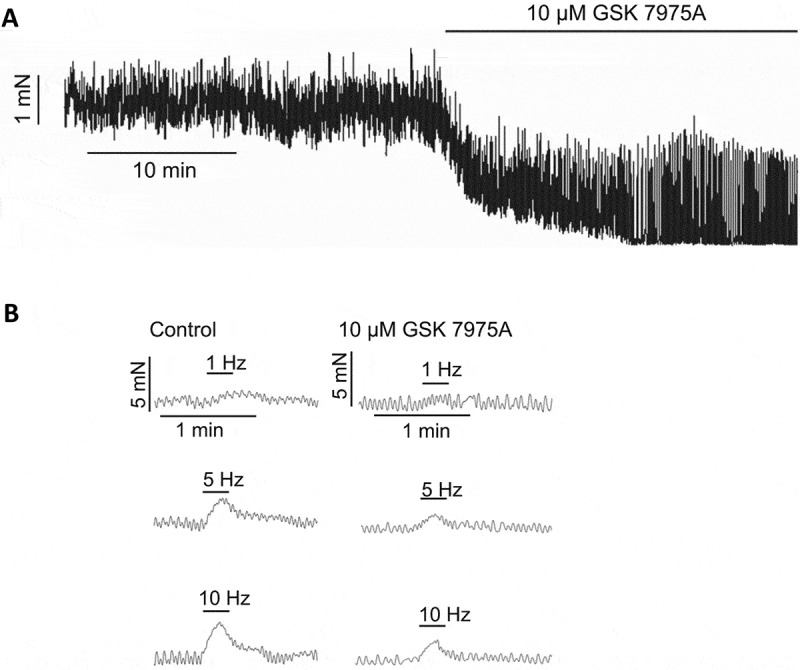
SOCE contributes to generation of tone and neurally evoked contractions of pig USM. A. Orai channel inhibitor GSK-7975A reduces myogenic tone of pig urethral smooth muscle measured by isometric tension. B. Contractions of pig urethral smooth muscle evoked by electrical field stimulation are reduced by GSK-7975A (adapted with permission from [25]).

In pig USM, GSK-7975A dramatically reduced myogenic tone by >50% (Figure 4(A)), and phenylephrine or electrical field stimulation evoked contractions by 40% (Figure 4(B)) [25]. Interestingly, nifedipine did have significant effects on the SOCE overshoot and agonist responses in pig USM, suggesting that unlike in mouse USMC, L-type Ca^2+^ channels and SOCE via Orai both contribute to contractility in pig USM, whereas mouse USMC appear to solely rely on SOCE to sustain activity under normal conditions. It could be that while one influx pathway serves as the primary Ca^2+^ influx mechanism in pig USM under most circumstances, the other may serve as a safety factor to sustain Ca^2+^ influx when the dominant pathway is impeded. A similar proposed mechanism occurs in airway smooth muscle, where effects of Orai or L-type channel inhibitors (GSK-7975A and nifedipine) exhibit moderate effects on cholinergic responses (evoked Ca^2+^ transients and contractions) in isolation, but GSK-7975A and nifedipine both abolish responses when the other pathway is inhibited [62].

## Possible mechanisms of SOCE contribution to in USMC Ca^2+^ signaling and contractility

**Figure 5.**
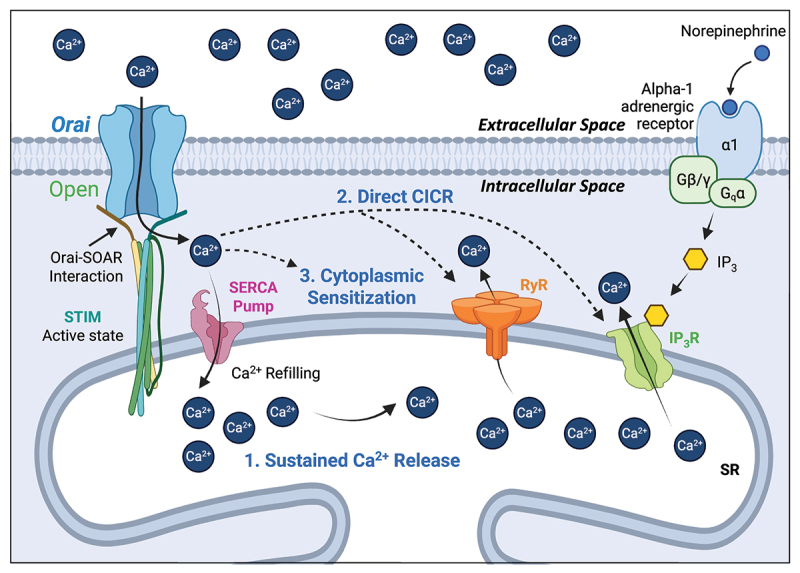
Possible mechanisms of SOCE to sustain Ca^2+^ signals and contractions of USMC. There are several possible mechanisms by which SOCE mediated by Orai channels may affect USMC contractility by enhancing USMC Ca^2+^ release that underlies contractions in these cells. 1) Orai mediated SOCE may refill SR Ca^2+^ stores via SERCA pumps, to enable sustained Ca^2+^ release via IP_3_Rs and RyRs. 2) Orai mediated SOCE may either directly initiate or amplify Ca^2+^ signals from IP_3_Rs and RyRs via Ca^2+^ induced Ca^2+^ release (CICR). 3) Orai mediated SOCE may raise cytoplasmic Ca^2+^ levels to sensitize IP_3_Rs or RyRs to more readily open and release Ca^2+^.

The evidence presented in this review suggests that SOCE mediated by Orai channels affect USMC contractility by contributing to Ca^2+^ release in USMC. Ca^2+^ release events in USMC are associated with contractions of USMC bundles and thus increased Ca^2+^ release would facilitate contraction. While a comprehensive explanation for how this occurs at the cellular level is currently lacking, we put forward three possible mechanisms by which this might occur, summarized in Figure 5. 1) Orai mediated SOCE may refill SR Ca^2+^ stores via canonical SERCA pathways, this would maintain a sufficiently large SR Ca^2+^ store load to enable sustained regenerative Ca^2+^ release via IP_3_Rs and RyRs. 2) Orai mediated SOCE may directly act on SR Ca^2+^ release channels such as IP_3_Rs and RyRs to either initiate or amplify Ca^2+^ signals via Ca^2+^ induced Ca^2+^ release (CICR). 3) Orai mediated SOCE may raise cytoplasmic Ca^2+^ levels to create an excitable medium for SR Ca^2+^ release and while not activating IP_3_Rs or RyRs directly, this process may sufficiently sensitize them to open in response to an initial localized Ca^2+^ signal from the SR release channels. These possibilities are not exclusive and there could be combinations or crosstalk between them. Thus, as all of these potential pathways would enhance Ca^2+^ release in USMC, which is the underling mechanism that underpins USMC contractility, this provides possible means for SOCE to influence urethral contractility functionally.

## SOCE in urethral interstitial cells

**Figure 6.**
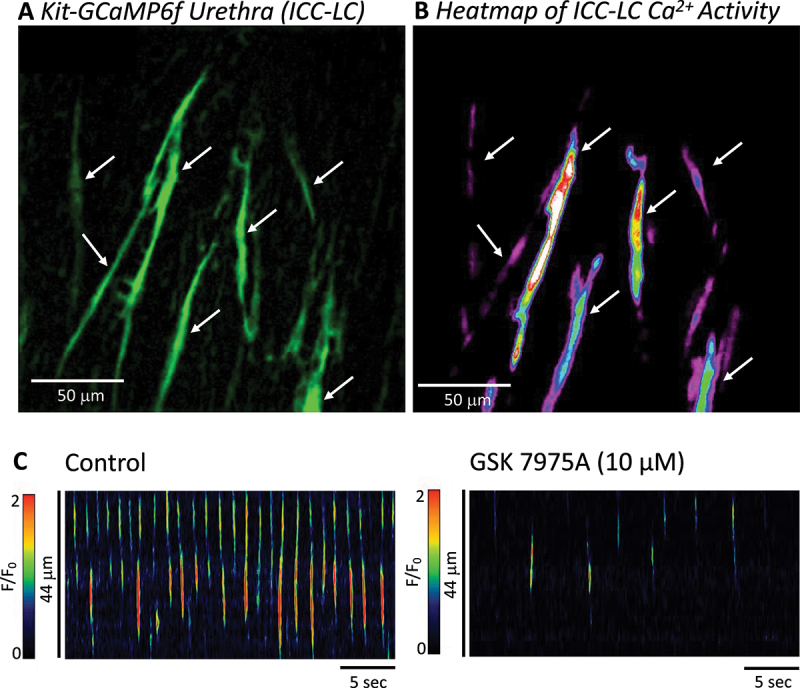
Spontaneous activity of mouse urethral interstitial cell of Cajal-like cells relies on SOCE. A. The internal urethral sphincter contains interstitial cells termed ICC-LC (interstitial cell of Cajal-like cells) that are morphologically and functionally distinct from urethral smooth muscle cells. Note the elongated and sometimes “spiny” morphology of ICC-LC (imaged with anti-GFP antibodies from mice expressing the Ca^2+^ indicator GCaMP6f exclusively in Kit^+^ cells). B. Heatmap of Ca^2+^ activity in ICC-LC over a 20 sec recording demonstrating ICC-LC are spontaneously active. C. STMs from an ICC-LC in mouse urethra demonstrate that the Orai channel inhibitor GSK-7975A reduces activity (adapted with permission from [6]).

In addition to USMC, the walls of the urethra contain of interstitial cells (5–10% of total cellular population (Figure 6(A,B)) [77]) that lack smooth muscle contractile proteins, but display spontaneous electrical and Ca^2+^ signaling behaviors that may influence activity of USMC to which they are connected [78]. Owing to their morphological and proposed functional similarities to Kit^+^ interstitial cells of Cajal (ICC) in the gastrointestinal tract, which serve as smooth muscle pacemakers [79] and neuromodulators [80], this manuscript will collectively refer to such interstitial cells in the urethra as ICC-like cells (ICC-LC) [8].

In patch clamp recordings, rabbit urethral ICC-LC exhibit spontaneous depolarizations in current clamp and STICs in voltage clamp mode which are mediated by Ca^2+^-activated-Cl^−^ channels [77,81,82], encoded by ANO1 [83]. When imaged either in intact urethral tissues from mice, or from isolated cell suspensions from rabbit, urethral ICC-LC fire a continuum of Ca^2+^ transients, ranging from brief localized events to intracellular Ca^2+^ waves that propagate great lengths of the cell [6,68,84,85]. The propagation of Ca^2+^ waves, and subsequent activation of Ca^2+^-activated-Cl^−^ channels in rabbit ICC-LC relies on Ca^2+^ influx from the extracellular space [84,85]. Evidence for a possible role of SOCE in coordinating this urethral ICC-LC activity is currently not consistent, which may reflect differences in species under study across investigators.

Urethral ICC-LC have been most extensively studied at the single cell level from enzymatically dissociated rabbit tissues. Rabbit ICC-LC do possess a capacity for SOCE (termed capacitative Ca^2+^ entry in some publications), as demonstrated by a hallmark SOCE “overshoot” in Ca^2+^ influx recorded with fura-2 microfluorimetry under conditions of store depletion to evoke SOCE [86]. SKF-96365, a nonselective pharmacological SOCE inhibitor significantly reduced rabbit ICC-LC spontaneous intracellular Ca^2+^ wave frequency and La^3+^ abolished them [84], suggesting that SOCE could contribute to Ca^2+^ store refilling to sustain activity in ICC-LC. Curiously however, nonselective SOCE inhibitors such La^3+^ and Gd^3+^ (which effectively reduced the SOCE “overshoot” in rabbit ICC-LC by >60%) did not affect STICs recorded from these cells in voltage-clamp [86] suggesting that while ICC-LC had the capacity for SOCE, this pathway was not essential for spontaneous activity under basal conditions. The discrepancy of how La^3+^ had no effect on STICs recorded from ICC-LC but abolished Ca^2+^ waves in other ICC-LC was not resolved, and later experiments concluded that the primary Ca^2+^ influx pathway for sustaining spontaneous activity in rabbit urethral ICC-LC was reverse mode Na^+^-Ca^2+^ exchange (NCX) and not SOCE [85,87,88].

It is interesting to note that in gastrointestinal ICC, from which urethral ICC-LC share many commonalities (expression of tyrosine kinase marker Kit, similar morphology and proposed physiological function as smooth muscle pacemakers and neuromodulators), STIM–Orai interactions are critical for generation and maintenance of Ca^2+^ activity [60,89–93]. Using transgenic mice expressing the optogenetic Ca^2+^ sensor GCaMP6f expressed exclusively in Kit^+^ ICC and ICC-LC [94], a 2024 study revealed a population of murine urethral ICC-LC *in situ* with dynamic, non-coordinated spontaneous Ca^2+^ activity [6] similar to that previously recorded from rabbit [68]. In these preparations, GSK-7975A significantly reduced Ca^2+^ transient frequency by >80%, and reduced Ca^2+^ transient amplitude, duration, and spatial spread by >50% (Figure 6(C)), suggesting that Ca^2+^ influx via Orai channels was vital for this activity [6].

Disparities in consistent effects of SOCE inhibitors on ICC-LC in studies from mice and rabbits might be accounted for by differences in pharmacological tools (GSK-7975A vs SKF 96,365 or La^3+^). Alternatively, the relative importance of SOCE in ICC-LC might vary across species. Such species differences in urethral ion channel expression and function have been documented in other situations. For example, ICC-LC and not USMC exclusively express ANO1 channels in rabbit urethra [83], and ANO1 inhibitors significantly reduce urethral tone and neurally evoked contractions [83]. Conversely, murine ICC-LC do not express ANO1 channels, while USMC express ANO1 abundantly, and yet ANO1 inhibitors do not affect mouse urethral tone or contractions evoked by agonists or nerve stimulation [95]. It could be the case that while SOCE is evolutionarily conserved in ICC-LC across mice and rabbit (as both can demonstrate SOCE “overshoots” upon Ca^2+^ store depletion), different Ca^2+^ influx pathways might be required to sustain spontaneous activity (although to date the effect of NCX modulators on mouse ICC-LC activity has not been tested and thus it is unknown if these cells may utilize a combination of SOCE and NCX as Ca^2+^ influx pathways).

## Conclusions and avenues for future investigation

SOCE, mediated by STIM/Orai interactions is a key mechanism for sustaining the spontaneous activity of USMC in animal models, although the importance of the pathway to generate functional contractile responses varies among species. As described above, possible species differences in the capacity of USMC to utilize SOCE for their contractile functions need to be investigated more fully, and studies of human USMC are currently lacking. In addition to species differences, variability in sex should be considered. Murine USMC display numerous sex differences in contractile properties due to differing ion channel and receptor expression profiles [96,97]. To date, evidence for Orai mediated SOCE impacting USMC activity in mice has been derived solely from male animals [7,76], and experiments on rabbits and pigs were not separated based on sex, meaning that any sex-dependent variations were not examined. A clear comparative study of SOCE function in male vs female models is therefore warranted to preclude sex as a biological variable in evaluating SOCE and STIM/Orai channels as viable target for urinary incontinence therapies across males and females.

Upregulation of STIM and/or Orai is associated with several smooth muscle pathologies, such as airway hyperresponsiveness and asthma [98,99], erectile dysfunction [100] and hypertension/vascular remodeling [101]. In such diseases, upregulated STIM/Orai expression leads to hypercontractile smooth muscle due to excessive Ca^2+^ oscillations that activate contractile machinery [98], or upregulated Ca^2+^ activation of transcription factors that induce remodeling and proliferation of smooth muscle [98,101–103]. Whether elements of SOCE machinery are also upregulated in urethral hypercontractile or remodeling pathophysiological states that manifest as obstruction disorders (bladder outlet obstruction or benign prostatic hyperplasia) is currently unknown and is worthy of study. There is also no information currently on whether SOCE leads to activation of genetic pathways in USMC of any species, or whether SOCE sole role is to facilitate cytoplasmic/SR Ca^2+^ levels for Ca^2+^ transient firing in USMC, this should be studied in future investigations.

## Data Availability

All data presented in this paper is available from the corresponding author upon reasonable request.
